# Thiolation of Chitosan Loaded over Super-Magnetic Halloysite Nanotubes for Enhanced Laccase Immobilization

**DOI:** 10.3390/nano10122560

**Published:** 2020-12-20

**Authors:** Avinash A. Kadam, Bharat Sharma, Surendra K. Shinde, Gajanan S. Ghodake, Ganesh D. Saratale, Rijuta G. Saratale, Do-Yeong Kim, Jung-Suk Sung

**Affiliations:** 1 Research Institute of Biotechnology and Medical Converged Science, Dongguk University-Seoul, Ilsandong-gu, Goyang-si, Gyeonggi-do, Seoul 10326, Korea; kadamavinash@dongguk.edu (A.A.K.); rijutaganesh@gmail.com (R.G.S.); 2 Department of Materials Science and Engineering, Incheon National University, Academy Road Yeonsu, Incheon, Seoul 22012, Korea; bharatsharma796@gmail.com; 3 Department of Biological and Environmental Science, Dongguk University-Seoul, Ilsandong-gu, Goyang-si, Gyonggido, Seoul 10326, Korea; surendrashinde.phy@gmail.com (S.K.S.); ghodakegs@gmail.com (G.S.G.); 4 Department of Food Science and Biotechnology, Dongguk University-Seoul, Ilsandong-gu, Goyang-si, Gyeonggi-do, Seoul 10326, Korea; gdsaratale@gmail.com; 5 Department of Life Science, College of Life Science and Biotechnology, Dongguk University-Seoul, Ilsandong-gu, Goyang-si, Gyonggido, Seoul 10326, Korea

**Keywords:** laccase, immobilization, thiolated chitosan, super-magnetic nanosupports for enzyme immobilization, ampicillin, Direct Red 80

## Abstract

This study focuses on the development of a nanosupport based on halloysite nanotubes (HNTs), Fe_3_O_4_ nanoparticles (NPs), and thiolated chitosan (CTs) for laccase immobilization. First, HNTs were modified with Fe_3_O_4_ NPs (HNTs-Fe_3_O_4_) by the coprecipitation method. Then, the HNTs-Fe_3_O_4_ surface was tuned with the CTs (HNTs-Fe_3_O_4_-CTs) by a simple refluxing method. Finally, the HNTs- Fe_3_O_4_-CTs surface was thiolated (-SH) (denoted as; HNTs- Fe_3_O_4_-CTs-SH) by using the reactive NHS-ester reaction. The thiol-modified HNTs (HNTs- Fe_3_O_4_-CTs-SH) were characterized by FE-SEM, HR-TEM, XPS, XRD, FT-IR, and VSM analyses. The HNTs-Fe_3_O_4_-CTs-SH was applied for the laccase immobilization. It gave excellent immobilization of laccase with 100% activity recovery and 144 mg/g laccase loading capacity. The immobilized laccase on HNTs-Fe_3_O_4_-CTs-SH (HNTs-Fe_3_O_4_-CTs-S-S-Laccase) exhibited enhanced biocatalytic performance with improved thermal, storage, and pH stabilities. HNTs-Fe_3_O_4_-CTs-S-S-Laccase gave outstanding repeated cycle capability, at the end of the 15th cycle, it kept 61% of the laccase activity. Furthermore, HNTs-Fe_3_O_4_-CTs-S-S-Laccase was applied for redox-mediated removal of textile dye DR80 and pharmaceutical compound ampicillin. The obtained result marked the potential of the HNTs-Fe_3_O_4_-CTs-S-S-Laccase for the removal of hazardous pollutants. This nanosupport is based on clay mineral HNTs, made from low-cost biopolymer CTs, super-magnetic in nature, and can be applied in laccase-based decontamination of environmental pollutants. This study also gave excellent material HNTs-Fe_3_O_4_-CTs-SH for other enzyme immobilization processes.

## 1. Introduction

Environmental pollution is a daunting challenge to face in the modern world [1]. Pollutants from land and water are foremost in reducing the quality of life and human health [2]. Textile dyes released from textile industries into the water lead to several hazards to the environment, mainly due to their recalcitrant and toxic nature [3]. In recent decades, emerging pharmaceutical contaminants released in very small concentrations caused a significant challenge to the environment, mainly due to their bioactive nature and the development of antibiotic resistance in the microbes [4]. The pharmaceutical compounds caused the development of antibiotic resistance in the microbes, hence taking the entire world into a serious health challenge [5]. Various methods and combinations of treatment were employed to face this challenge [6]. However, enzymatic methods unveiled a new outlook to treat waste streams to get rid of intractable organic pollutants [7]. Laccase is one of the most important enzymes in terms of waste pollutant removal. Laccase catalyzes one-electron oxidation of the pollutant using molecular oxygen as the electron donor [8]. However, free laccase has several disadvantages: its proteinaceous nature, it is more prone to denaturation, stability issues, and higher cost due to use in a single reaction [9]. The immobilization approach will cover all of these disadvantages and provide a more suitable and accessible biocatalyst. Therefore, suitable, novel, and efficient nanosupports for the immobilization of laccase are required for its potential applications in the remediation of environmental pollutants [1].

Several supports have been reported for laccase immobilization [10]. Of the nanosupports studied for immobilization, the naturally available clay mineral halloysite nanotube (HNT) is catching significant attention. This is mainly due to a number of important properties of HNTs [11]. These include: easy and low-cost availability, structural morphology in the form of nanotubes, nanotubular lumen, and an extremely modifiable surface [12]. The surface modifications of HNTs are the main key to developing them into a comprehensive nanosupport for laccase immobilization [13,14]. The surface modification of HNTs with Fe_3_O_4_ nanoparticles (NPs) makes HNTs super-magnetic. This adds the important parameter of magnetic separation [15]. Being HNT-immobilized, laccase separation from solution is an extremely important parameter for laccase recovery and enhanced laccase applications [16]. Furthermore, the surface of the super-magnetic HNTs can be modified with a biopolymer like chitosan (CTs) [17]. As CTs is a ubiquitous biopolymer used for enzyme immobilization due to having the surface amino (-NH_2_) functional group. In our previous research, modification of the HNT surface with Fe_3_O_4_ and CTs proved to be excellent for laccase immobilization [13,14,18]. In these studies, the immobilization of “laccase” and “CTs and Fe_3_O_4_ modified HNTs” was carried out by glutaraldehyde cross-linking. Still, there is a huge scope to adopt new immobilization strategies. One such important strategy is thiolation (-SH) of the amino (-NH_2_) group of CTs loaded on magnetic-HNTs [19,20,21]. Therefore, this study mainly emphasized the thiolation of the amino (-NH_2_) group present of CTs-modified magnetic HNTs.

Thiolation of chitosan has attracted significant attention in recent years for many desirable applications such as enzyme immobilization, drug delivery, cosmetics, and tissue engineering [19,20]. Thiolation of the CTs involves modification of the surface amino (-NH_2_) group to the thiol (-SH) group by NHS-ester reaction [22]. The enzymes possess the –SH group due to the presence of amino acids such as cysteine and methionine [7]. The –SH group from the thiolated supports and enzyme react at pH 5 to form a very stable (–S-S–) disulfide bond for immobilization [23]. Thus, thiolation of the CTs loaded over magnetized HNTs can provide highly applicable nanosupports for enzyme immobilization applications. As per the author’s literature survey, thiolated CTs, Fe_3_O_4,_ and HNT nanocomposite has not yet been reported for enzyme immobilization.

Thus, in summary, this study attempts the synthesis of nanocomposites mainly containing HNTs, Fe_3_O_4_ NPs, and thiolated CTs for laccase immobilization and application of the newly synthesized immobilization system for the biocatalytic degradation of the environmental pollutants Direct Red 80 (DR80) and the pharmaceutical compound ampicillin. The structural and morphological details of the nanocomposites were assessed with various techniques such as; FE-SEM, HR-TEM, XPS, FT-IR, and XRD analyses. The biocatalysis of immobilized laccase was carried out in detail. The degradation of DR80 and ampicillin were checked by the developed immobilized system.

## 2. Materials and Methods

### 2.1. Materials

Halloysite nanotubes (nanopowder), ammonium hydroxide (ACS Grade Solvents, 25%, NH_4_OH), chitosan (low molecular weight), N-(3-dimethylaminopropyl)-N′-ethylcarbodiimide hydrochloride (≥98% (titration), EDAC.HCl), N-hydroxysuccinimide (≥97.0% (titration) NHS), thioglycolic acid (≥98%), N,N-dimethylformamide (99.8% DMF), ampicillin (anhydrous), Direct Red 80 (powder), syringaldehyde (SA, assay- ≥ 98%), guaiacol (GUA, assay- ≥ 98%), p-Coumaric acid (CA, ≥98.0% high-performance liquid chromatography (HPLC)), 1-Hydroxybenzotriazole hydrate (HBT, ≥97.0% (T)), 2,2-Azino-bis(3-ethylbenzothiazoline-6-sulfonic acid) diammonium (ABTS, Liquid Substrate System), methanol (HPLC grade), acetonitrile (HPLC grade), water (HPLC grade) and laccase from *Trametes Versicolor* (powder) were obtained from the Sigma Aldrich, St. Louis, MO, USA. FeCl_3_-6H_2_O and FeCl_2_·4H_2_O were received from JUNSEI (Kyoto) Japan.

### 2.2. Synthesis of HNTs-Fe_3_O_4_-CTs-SH

The HNTs-Fe_3_O_4_-CTs-SH was synthesized in three steps. First, the HNTs were tuned with Fe_3_O_4_ NPs by the coprecipitation method [13]. Second, Fe_3_O_4_ NP-modified HNTs (HNTs-Fe_3_O_4_) were functionalized with the chitosan (CTs) (HNTs-Fe_3_O_4_-CTs) [14]. Finally, in the third step, NH_2_ functional groups of HNTs-Fe_3_O_4_-CTs were thiolated (-SH) by NHS-ester reaction (HNTs-Fe_3_O_4_-CTs). In the first step, 0.2–2 g of HNTs was added to 200 mL of deionized water. The mixture was ultrasonicated by Sonics Vibra-Cell VC130 Ultrasonic Processor, power—130 W, frequency—20 kHz, and amplitude—60 µM (Sonics & Materials, Inc., Newtown, CT, USA) for 1 h. This mixture was added to 100 mL of 2% FeCl_3_·6H_2_O, and 100 mL of 1% FeCl_2_·4H_2_O (dropwise) in the presence of N_2_ gas at 60 °C under constant stirring. Then, 30 mL of 25% NH_4_OH solution was added to the mixture. The obtained black colored precipitate of nanocomposite was stirred at 60 °C for 1 h. Further, it was separated magnetically, washed thoroughly with water, ethanol, and methanol, and dried in an oven at 60 °C for 48 h. The obtained sample of HNTs-Fe_3_O_4_ was powdered in a mortar and pestle for further use.

In the second step, the CTs modification of HNTs-Fe_3_O_4_ was done. In a typical reaction, the CTs (1 g) was taken in 100 mL of 1% acetic acid and vortexed for 4 h on the magnetic stirrer adjusted to the 50 °C. The CTs solution was prepared in 1% acetic acid in warm conditions to obtain a clear solution of completely dissolved CTs. Then 1 g of HNTs-Fe_3_O_4_ was taken in 100 mL distilled water and ultrasonicated with Sonics Vibra-Cell VC130 Ultrasonic Processor, power—130 W, frequency—20 kHz, and amplitude—60 µM (Sonics & Materials, Inc., Newtown, CT, USA) for 1 h. Next, both the CTs solution and ultrasonicated HNTs-Fe_3_O_4_ solution were poured into a 500 mL beaker and stirred for 15 min. At this point, 2 mL of glutaraldehyde solution (2.5%) was added to the mixture. Further, the beaker was covered with the aluminum foil and continued the stirring for 8 h on the magnetic stirrer adjusted to 50 °C. The obtained HNTs-Fe_3_O_4_-CTs nanocomposite was separated magnetically, washed thoroughly with distilled water, ethanol, and methanol, dried in an oven at 60 °C for 48 h, and finally powdered for further use.

Further, the thiolation of the HNTs-Fe_3_O_4_-CTs was done with the NHS-ester reaction. In a typical reaction, EDAC (6.08 mM), NHS (5.79 mM), and 5 mL of TGA were added to the 10 mL of the DMF. The mixture was kept in shaking conditions of 200 rpm for 24 h at 25 °C to form a reactive NHS-ester. Then, the HNTs-Fe_3_O_4_-CTs and NHS-ester reaction were carried out to form a thiol functionalized HNTs-Fe_3_O_4_-CTs (HNTs-Fe_3_O_4_-CTs-SH). In this typical reaction, HNTs-Fe_3_O_4_-CTs (6.6 g/L) was taken in pH 5.0 buffer (Na-acetate buffer 100 mM) and further added with the NHS-ester (0.67 mL/L) in dark conditions. The mixture was vortexed rapidly at 200 rpm and 25 °C for 4 h in dark conditions. Once the reaction was completed, the materials were immediately removed magnetically and washed thoroughly with pH 5.0 buffer (Na-acetate buffer 100 mM). The obtained materials were dried in a freeze dryer and powdered for further use for enzyme immobilization study.

### 2.3. Laccase Immobilization Experiment

The laccase immobilization experiment was carried out in a 20 mL glass tube. The materials HNTs-Fe_3_O_4_-CTs and HNTs-Fe_3_O_4_-CTs-SH (1 g/L) were taken in 20 mL glass tubes separately. Each of these tubes was added with 10 mL of laccase solution (1.5 g/L) prepared in the 100 mM Na-acetate buffer (pH-4). These mixtures were immediately kept shaking (200 rpm) and at a temperature of 25 °C for 24 h. After completion of the immobilization process, the materials were magnetically removed and washed thoroughly with the 100 mM Na-acetate buffer (pH-4) to remove all unbound laccase. The laccase immobilized materials were then tested for activity recovery (%) and laccase-loading capacities. Laccase activity was determined in the 2 mL reaction mixture having 0.9 mL of sodium acetate buffer (100 mM, pH 4), 1 mL of ABTS solution (90 µM), and free laccase (0.1 mL from 1.5 mg/mL of laccase solution) or immobilized laccase (0.1 mL solution containing 1 mg of HNTs-Fe_3_O_4_-CTs-S-S-Laccase). The reaction mixtures of both free laccase and immobilized laccase were kept gently mixing for 30 min. ABTS oxidation was quantified by the absorption at 420 nm for ABTS (molar extinction coefficient [ε420], 0.0360 µM^−1^ cm^−1^). The 1 U of laccase activity was measured as the ABTS (µM) oxidation per min of incubation time and per mL of solution. The detailed formulae of the measurements of activity recovery (%) are given by Equation (1) [13].

(1) $$Activity\;recovery\;\left(\%\right)\;=\;A_{IL}/A_{FL}\times 100$$

where *A_IL_* is the immobilized laccase activity, and *A_FL_* is the free laccase activity before the immobilization procedure. The laccase loading capacity (mg/g) was determined by the Bradford method (Add reference) using the Pierce Coomassie (Bradford) Protein Assay Kit, Thermo Scientific, Massachusetts, MA, USA. The detailed formulae of the measurements of laccase loading capacity are given by Equation (2) [13].

(2) $$Laccase\;loading\;(mg/g)\;=\;\left(C_{bi}-C_{ai}\right)V/W$$

where *C_bi_* is laccase concentration before immobilization (mg/L), *C_ai_* is retained laccase concentration in solution after immobilization (mg/L), *V* is the volume of the solution in liters (L), and *W* is the weight of the nanocomposites in grams (*g*). Further, the immobilized laccase was analyzed for biocatalytic properties.

### 2.4. Characterizations

The modified materials were morphologically characterized by scanning electron microscopy (SEM, FC-SM10, Hitachi S-4800, Ibaraki, Japan) and high-resolution transmission electron microscopy (HR-TEM, Tecnai G2 transmission electron microscope, Hillsboro, OR, USA). The samples for SEM and TEM were prepared in 10 mL distilled water containing 10 mg of each sample. The samples were ultrasonicated (Sonics Vibra-Cell VC130 Ultrasonic Processor (Sonics & Materials, Inc., Newtown, CT, USA) for 1 h. The well-dispersed samples were drop coated on TEM carbon grid and silicon wafer for SEM and TEM analysis, respectively. The crystalline purity of the samples (in powder form) were analyzed by X-ray powder diffractometer (XRD, Cu-Kα radiation (λ = 1.5418 Å), Ultima IV/Rigaku, Tokyo, Japan). The magnetic properties of samples (in powder form) were analyzed by vibrating sample magnetometer (VSM, Lakeshore, Model: 7407, LA, USA). The functional group profile of samples (in powder form) were analyzed by Fourier transform infrared spectroscopy (FT-IR, Spectrum 100, PerkinElmer, Waltham, MA, USA). The surface elemental profile of the samples (in powder form) were analyzed by X-ray photoelectron spectroscopy (XPS, Theta Probe AR-XPS System, Thermo Fisher Scientific, Dartford, UK).

### 2.5. Biocatalysis of Immobilized Laccase

The effect of the initial laccase concentration on laccase loading on HNTs-Fe_3_O_4_-CTs-SH was assessed by taking laccase concentrations of 0.5, 0.75, 1.0, 1.25, 1.5, and 1.75 mg/mL. The laccase loading was evaluated as per the Equation (2) from Section 2.3. The temperature stability was analyzed by incubating the free laccase and immobilized laccase in acetate buffer (100 mM, pH 4.2) for 200 min at 60 °C. The samples were withdrawn after each 40 min interval and analyzed for relative activity (%). The relative activity was calculated by considering the initial activity before the stability experiment as 100% [13]. The calculation of relative activity is given by the following formulae Equation (3).

(3) $$Relative\;activity\;\left(\%\right)\;=\;A_{e}/A_{i}\;\times 100$$

where *A_e_* is the activity after the stability experiment, and *A_i_* is the initial activity before the stability experiment. Further, the storage stability was evaluated by incubating free and immobilized laccase acetate buffer (100 mM, pH 4.2) for 30 days at 4 °C. The sample was tested for relative activity (%) after every 5 days. The pH stability was also evaluated by incubating the free and immobilized laccase at various pH 1–9 for 1 h and at a temperature of 20 °C. The samples were measured for the relative activity (%) after the incubation process. The reusability experiments of immobilized laccase were carried out by magnetically removing the immobilized laccase after completion of the first reaction cycle, and this was followed by the addition of the new reaction mixture for the next cycle. This was continued for 15 cycles of reactions by the same immobilized laccase.

### 2.6. Immobilized Laccase Mediated Degradation of Environmental Pollutants

The target pollutants used were DR80 and ampicillin. Their degradation experiment was carried out in a typical reaction mixture that included acetate buffer (100 mM, pH 4.2), pollutant (DR80 (15 ppm)/ampicillin (25 ppm)), 1 mM redox mediators, and immobilized laccase/free laccase (0.1 mL) for 4 h, with a shaking condition of 200 rpm and temperature of 20 °C. The DR80 degradation was calculated by performing UV−vis spectroscopic analysis. The high-performance liquid chromatography (HPLC, Shimadzu LC-20AD, Kyoto, Japan) analysis was carried out for assessment of the ampicillin degradation. The HPLC analysis used the following parameters such as detection wavelength (230 nm), a mobile phase of [A:B:C:D] [A] acetonitrile, [B] water, [C] 1M potassium phosphate monobasic in water, [D] 1N acetic acid in water (80:909:10:1), the flow rate of 0.6 mL/min and the C18 column (Ascentis Express 90 Å, C18 10 cm × 4.6 mm, 5 µm, Sigma-Aldrich, St. Louis, MO, USA). The repeated cycle degradation of DR80 was carried out as follows, after the completion of the first degradation cycle as mentioned above, the immobilized laccase was magnetically removed, washed with sodium acetate buffer pH 4.2. This washed immobilized laccase was added to the fresh reaction mixture which included fresh acetate buffer (100 mM, pH 4.2), 1 mM redox mediator, and DR80 (15 ppm) for the next cycle. Similarly, 10 cycles were carried out to assess the reusability potential.

## 3. Results and Discussion

### 3.1. Synthesis

**Figure 1:**
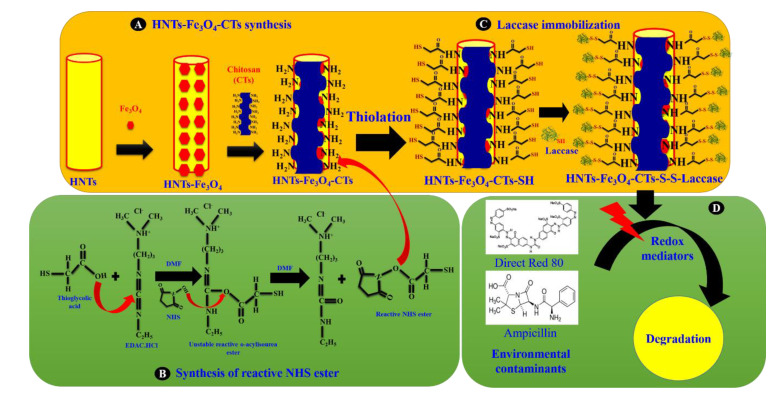
The schematic presentation of the synthesis of(**A**) HNTs-Fe_3_O_4_-CTs, (**B**) reactive NHS ester, and (**C**) HNTs-Fe_3_O_4_-CTs-S-S-Laccase, and (**D**) degradation of the environmental contaminants Direct Red 80 and ampicillin by the immobilized laccase.

The synthesis of HNTs-Fe_3_O_4_-CTs, reactive NHS ester, and HNTs-Fe_3_O_4_-CTs-S-S-Laccase are presented in Figure 1A–C. The pristine HNTs were first tuned with Fe_3_O_4_ by the coprecipitation method (Figure 1A). Further, the HNTs-Fe_3_O_4_ was modified with the CTs (Figure 1A) in a simple reaction process. It is well known that CTs exhibits ubiquitous NH_2_ groups on its surface, and this can be essential for thiolation. The process of thiolation involves modification of the materials with the thio (-SH) group by using the reactive NHS ester. The detailed synthesis of the reactive NHS ester was presented in Figure 1B. In the reaction, the thioglycolic acid reacted with EDAC.HCl to form an unstable reactive o-acylisourea ester. The NHS attached to the unstable o-acylisourea ester and formed the reactive NHS ester. In the next reaction of NHS ester with HNTs-Fe_3_O_4_-CTs, NHS ester attaches to the amino (NH_2_) functional group present on the surface of the HNTs-Fe_3_O_4_-CTs. This transforms the thiol moiety on the surface of the HNTs-Fe_3_O_4_-CTs (denoted as; HNTs-Fe_3_O_4_-CTs-SH). The reaction at pH 5 is very important for the reaction to occur. A similar mechanism for thiolated chitosan was explained by Hanif et al., 2015 [21]. The thiolated and CTs/Fe_3_O_4_ modified HNTs were applied for the laccase immobilization. The thiol group from the nanocomposite reacted with the thiol group from the laccase to form the strong disulfide (-S-S-) bond for immobilization. The thiol group displayed excellent immobilization performance for the enzyme laccase [7].

The laccase immobilized HNTs-Fe_3_O_4_-CTs-SH is denoted as HNTs-Fe_3_O_4_-CTs-S-S-Laccase (Figure 1C). Finally, after laccase immobilization, the HNTs-Fe_3_O_4_-CTs-S-S-Laccase was applied for the redox-mediated degradation of the textile dye Direct Red 80 (DR80) and pharmaceutical compound ampicillin (Figure 1D).

### 3.2. Characterizations

#### 3.2.1. SEM and HR-TEM Analysis

**Figure 2:**
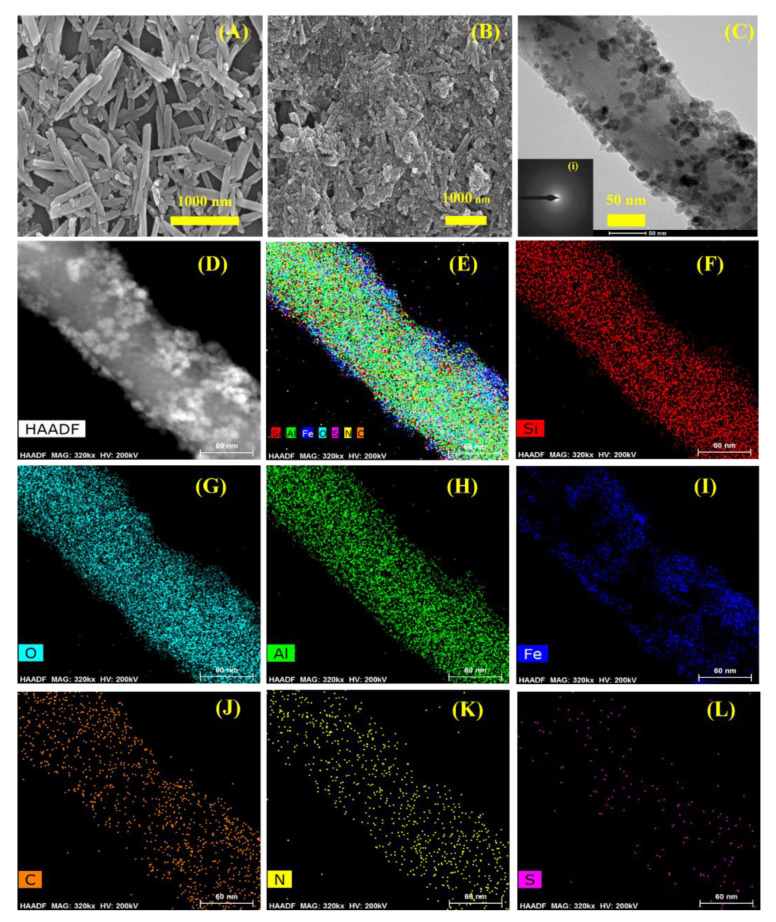
SEM analysis of (**A**) HNTs, and (**B**) HNTs-Fe_3_O_4_-CTs-SH. HR-TEM analysis of the HNTs-Fe_3_O_4_-CTs-SH (**C**) inset (i) SAED pattern of the HNTs-Fe_3_O_4_-CTs-SH. The STEM HAADF image of HNTs-Fe_3_O_4_-CTs-SH (**D**), elemental mapping of HNTs-Fe_3_O_4_-CTs-SH (**E**), and corresponding elemental distribution of Si (**F**), O (**G**), Al (**H**), Fe (**I**), C (**J**), N (**K**) and S (**L**).

The morphological observations of the modified material were done by SEM analysis as shown in Figure 2A,B. Figure 2A shows the unmodified HNTs. The image represents the diverse sized nanotubes with the plane and unmodified surface. The ends of the tubes were found open. Figure 2B shows the modified form of the HNTs, i.e., HNTs-Fe_3_O_4_-CTs-SH. A close look reveals that the surface of the HNTs was heavily modified with the subsequent modifications done on the pristine HNTs; such as Fe_3_O_4_, CTs, and thiolation. The tubes seemed to be broadened, possibly due to the coatings of the CTs. Similar observations for chitosan modified HNTs was seen in an earlier report [13,14]. The SEM morphologies gave an idea of the significant modification of the plane surface of the HNTs. Furthermore, it was very important to analyze the morphological observations in more detail. To achieve this, the HR-TEM analysis of the HNTs-Fe_3_O_4_-CTs-SH was carried out. Figure 2C shows the HR-TEM image of the HNTs-Fe_3_O_4_-CTs-SH. The image represents the surface of a single nanotube. The surface of the tube showed the Fe_3_O_4_ NPs decorated over the tubular surface. The Fe_3_O_4_ NPs were found to be 5–10 nm in size. The shape of the Fe_3_O_4_ NPs was found to be circular and quasi-polyhedral. The selected area (electron) diffraction (abbreviated as SAED) analysis of HR-TEM image of HNTs-Fe_3_O_4_-CTs-SH is shown in Figure 2C(i). The SAED pattern revealed the polycrystalline nature resulting from HNTs and Fe_3_O_4_ NPs. Moreover, it was important to understand the subsequent modification on the nanotube with the evident elemental distribution. To observe this, high-angle annular dark-field imaging (HAADF) S-TEM analysis was carried out in Figure 2D,E. The combination of all the elements was revealed in Figure 2E.

**Figure 3:**
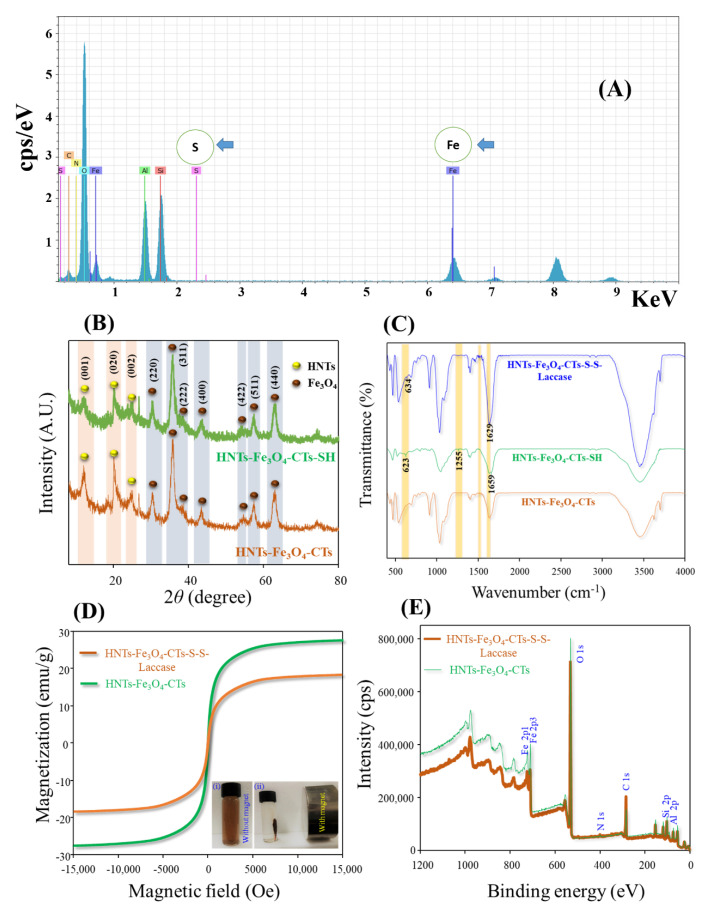
**(A**) HR-TEM EDS analysis of HNTs-Fe_3_O_4_-CTs-SH, (**B**) XRD analysis of HNTs-Fe_3_O_4_-CTs and HNTs-Fe_3_O_4_-CTs-SH, (**C**) FT-IR analysis of HNTs-Fe_3_O_4_-CTs, HNTs-Fe_3_O_4_-CTs-SH, and HNTs-Fe_3_O_4_-CTs-S-S-Laccase (**D**) VSM analysis of HNTs-Fe_3_O_4_-CTs and HNTs-Fe_3_O_4_-CTs-S-S-Laccase inset (**i**) HNTs-Fe_3_O_4_-CTs-S-S-Laccase without magnet, and (**ii**) HNTs-Fe_3_O_4_-CTs-S-S-Laccase with magnet, (**E**) XPS analysis of HNTs-Fe_3_O_4_-CTs and HNTs-Fe_3_O_4_-CTs-S-S-Laccase.

The element maps of silicon (Si), oxygen (O), aluminum (Al), iron (Fe), carbon (C), nitrogen (N), and sulfur (S) are shown in Figure 2F–L, respectively. The dense presence of Si, Al, and O elements outlined the basic backbone structure of the HNTs (Figure 2F–H). The presence of Fe represented the Fe_3_O_4_ NPs decorated over the tubular surface (Figure 2I). The presence of C and N designated the CTs modification (Figure 2J,K). Finally, the presence of the S over the nanotube confirmed the thiolation of the HNTs-Fe_3_O_4_-CTs (Figure 2L). Likewise, the elemental distribution was also confirmed by the HR-TEM energy-dispersive X-ray spectroscopy (EDS) analysis carried out to confirm the elemental distributions (Figure 3A). The presence of Fe, C, N, and S corroborated the successful modification of the HNT surface with the Fe_3_O_4_ NPs, CTs, and thiolation. The overall SEM and HR-TEM analysis gave the idea about the surface morphology, polycrystalline nature, and elemental distributions of the modified HNT materials.

#### 3.2.2. XRD, FT-IR, VSM, and XPS Analysis

The crystalline nature of HNTs-Fe_3_O_4_-CTs before and after thiolation was assessed using the XRD analysis, Figure 3B. The HNTs-Fe_3_O_4_-CTs and HNTs-Fe_3_O_4_-CTs-SH showed similar diffraction peak profiling. This revealed the intact crystalline pattern after the thiolation process. The typical diffraction peak patterning of HNTs, i.e., 11.94°, 20.33°, and 24.36° 2θ angles corresponding to the crystalline planes of (001), (020), and (002) was obtained in both the samples. Similar profiling of the HNTs peaks after modifications with Fe_3_O_4_ NPs and amino-salinization was reported [16]. The Fe_3_O_4_ NPs diffraction peaks were seen in both the samples at 30.35, 35.65, 37.46, 43.45, 54.54, 57.36, and 62.92° 2θ angles corresponding to the crystalline planes of the (220), (311), (222), (400), (422), (511), and (440), respectively, as per the JCPDS cards 75-0033 data [24]. Thus, the XRD analysis confirmed the Fe_3_O_4_ NPs modification over the HNTs.

The functional group profile of the HNTs-Fe_3_O_4_-CTs, HNTs-Fe_3_O_4_-CTs-SH, and HNTs-Fe_3_O_4_-CTs-S-S-Laccase was analyzed by the FT-IR analysis, shown in Figure 3C. The absorption peaks obtained in all the samples at 1028 and 910 cm^−1^ corresponded to siloxane vibration and silanol vibration, respectively [16]. The absorption peaks obtained in all the samples at 472 cm^−1^ corresponded to the Fe-O bond from Fe_3_O_4_ NPs [9]. The absorption peaks of 3448, 1550, 1443, 1322, and 1119 cm^−1^ obtained in all the samples, corresponded to the O–H stretching, N–H deformation, C–H deformation, C–N vibrations, and C–OH stretching of the CTs [9]. Hence, all the samples gave a similar peak profile corresponding to the peaks of HNTs, Fe_3_O_4,_ and CTs. This corroborates the successful synthesis of the HNTs-Fe_3_O_4_-CTs. However, the additional peaks of 1659, 1255, and 623 cm^−1^ were observed in the HNTs-Fe_3_O_4_-CTs-SH. These peaks mainly corresponded to the C=O stretching amides, C–SH stretching, and C–S stretching, respectively [7,25]. The appearance of these peaks successfully corroborated the thiolation of the chitosan. However, in the spectra of HNTs-Fe_3_O_4_-CTs-SH, the S-H stretch in the region of 2600–2550 cm^−1^ was absent. A similar observation for the absence of S-H stretch in the region of 2600–2550 cm^−1^ for thiolated chitosan was observed by an earlier report [7]. This might be due to selective thiolation rather than thiolation of most of the -NH_2_ group of the chitosan. Moreover, the FT-IR analysis of the HNTs-Fe_3_O_4_-CTs-S-S-Laccase is shown in Figure 3C. The peak corresponding to the disulfide bond was obtained at the 634 cm^−1^ [26,27]. The presence of the absorption peak at 1629 cm^−1^ corresponds to the amide II from proteins [28]. These all observations obtained in the FT-IR spectra of the HNTs-Fe_3_O_4_-CTs-S-S-Laccase indicated the successful loading of the laccase over the Fe_3_O_4_-CTs-SH through the disulfide covalent bond. The obtained results of the FT-IR are in strong agreement with the HAADF STEM elemental mapping and HR-TEM EDS analyses.

The magnetic potential of the materials played a crucial role in the separation mechanism. Hence, it was very important to know the magnetic properties of the synthesized material HNTs-Fe_3_O_4_-CTs and immobilized laccase HNTs-Fe_3_O_4_-CTs-S-S-Laccase. The magnetic properties of the HNTs-Fe_3_O_4_-CTs and HNTs-Fe_3_O_4_-CTs-S-S-Laccase were assessed by the VSM analysis (Figure 3D). The VSM analysis of both the samples exhibited the typical hysteresis curve of the magnetization with coercivity and remanence values to be zero. The magnetic potential value was observed to be 18.38 and 27.53 emu/g. All these observations were designated the superparamagnetic nature of both the samples. Besides, the magnetization potential of HNTs-Fe_3_O_4_-CTs-S-S-Laccase decreased with 9.15 emu/g in comparison with the HNTs-Fe_3_O_4_-CTs. This result confirmed the enhanced laccase loading over the surface of the modified material. Furthermore, the Figure 3D insets (i) and (ii) show the external magnet mediated separation of the HNTs-Fe_3_O_4_-CTs-S-S-Laccase from solution. All the VSM analyses confirmed the magnetic potential and laccase immobilization on materials.

**Figure 4:**
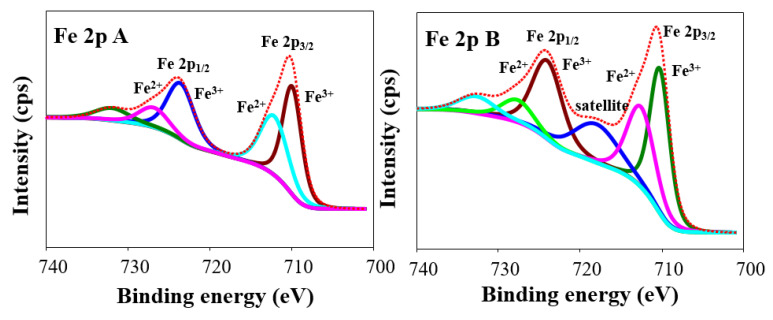
The high-resolution XPS spectrum of the Fe 2p peaks of (**A**) HNTs-Fe_3_O_4_-CTs, and (**B**) HNTs-Fe_3_O_4_-CTs-S-S-Laccase.

Furthermore, the surface elemental profile of the HNTs-Fe_3_O_4_-CTs and HNTs-Fe_3_O_4_-CTs-S-S-Laccase were examined by XPS analysis, Figure 3E. The HNTs-Fe_3_O_4_-CTs and HNTs-Fe_3_O_4_-CTs-S-S-Laccase showed the peaks Al 2p, Si 2p, C 1s, N 1s, O 1s, and Fe 2p at the binding energies of 74.88, 103.28, 284.60, 400.07, 532.41, and 710.47 eV, respectively. All the obtained elements confirmed the presence of Fe_3_O_4_ and CTs. However, the peaks of C1s from HNTs-Fe_3_O_4_-CTs-S-S-Laccase were found significantly higher in intensity than HNTs-Fe_3_O_4_-CTs. This corroborated the successful loading of the laccase. Additionally, the high-resolution spectra of Fe 2p for both the samples were examined with the curve fitting analysis (Figure 4). The close look at the curve fittings of Fe 2p showed two main peaks of Fe 2p1/2 and Fe 2p3/2 at the binding energies of the 710.42 and 723.80 eV, respectively. The spin energy separation of Fe 2p_1/2_ and Fe 2p_3/2_ was found to be 13.38 eV, which matches the standard data for Fe_3_O_4_ [29]. Iron in Fe_3_O_4_ exists in Fe^2+^ and Fe^3+^ oxidation states [30]. Hence, the Fe 2p curve fitting analysis showed both the states of Fe^2+^ and Fe^3+^ [31]. The spectra of both the samples were found to be split into Fe 2p_3/2_ and Fe 2p_1/2_ peaks at 710.4 and 724.0 eV corresponding to Fe^2+^, similarly Fe 2p_3/2_ and Fe 2p_1/2_ peaks of Fe^3+^ at 712.81 and 728.5 eV. However, the characteristic Fe satellite peak was observed in the HNTs-Fe_3_O_4_-CTs-S-S-Laccase. Similar Fe 2p peak profiling for Fe_3_O_4_-rGO was reported [32]. Thus, the overall XPS spectrum analysis corroborated the successful synthesis of modified HNTs and laccase immobilization.

### 3.3. Laccase Immobilization on HNTs-Fe_3_O_4_-CTs-SH

**Figure 5:**
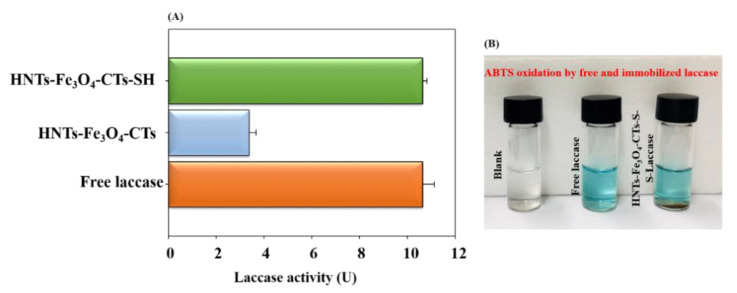
(**A**) Unit activity of free laccase (0.1 mL from 1.5 mg/mL laccase solution in sodium acetate buffer (100 mM, pH 4)), laccase immobilized on HNTs-Fe_3_O_4_-CTs (0.1 mL solution containing 1 mg of laccase immobilized HNTs-Fe_3_O_4_-CTs in sodium acetate buffer (100 mM, pH 4)), and HNTs-Fe_3_O_4_-CTs-S-S-Laccase (0.1 mL solution containing 1 mg of HNTs-Fe_3_O_4_-CTs-S-S-Laccase in sodium acetate buffer (100 mM, pH 4)). (**B**) Photographic image representing the color change after the oxidation of ABTS by free laccase and HNTs-Fe_3_O_4_-CTs-S-S-Laccase.

After structural and morphological confirmation of the HNTs-Fe_3_O_4_-CTs-SH synthesis, the HNTs-Fe_3_O_4_-CTs-SH was applied for immobilization of the laccase. The activity of free laccase, laccase immobilized on the HNTs-Fe_3_O_4_-CTs, and HNTs-Fe_3_O_4_-CTs-S-S-Laccase are shown in Figure 5A. The measure of 1 U of laccase activity is given as µM of ABTS oxidized/min/mL. The free laccase oxidized gave 10.60 U of the activity (Figure 5A). The laccase immobilized on HNTs-Fe_3_O_4_-CTs exhibited 3.3 U of the laccase activity (Figure 5A). This might be due to the adsorption of the laccase on HNTs-Fe_3_O_4_-CTs. However, the immobilization of laccase over HNTs-Fe_3_O_4_-CTs-SH (HNTs-Fe_3_O_4_-CTs-S-S-Laccase) possessed 10.64 U of the activity. Hence, HNTs-Fe_3_O_4_-CTs-S-S-Laccase exhibited 100% activity recovery (Figure 5A). Figure 5B shows the ABTS oxidized potential of free laccase and HNTs-Fe_3_O_4_-CTs-S-S-Laccase in photographic form. These results confirmed the significant enhancement in laccase activity after the thiolation of the support HNTs-Fe_3_O_4_-CTs. The thiol (-SH) group modification of chitosan provided unique covalent binding sites for the laccase immobilization. The thiol (-SH) group from the laccase covalently bound to the (-SH) group over the HNTs-Fe_3_O_4_-CTs-SH, to form the disulfide bond (-S-S-). This led to a significant enhancement of laccase loading over the HNTs-Fe_3_O_4_-CTs-SH. A similar mechanism of the laccase immobilization over the (-SH) modified supports was reported earlier [7,19,33,34].

**Figure 6:**
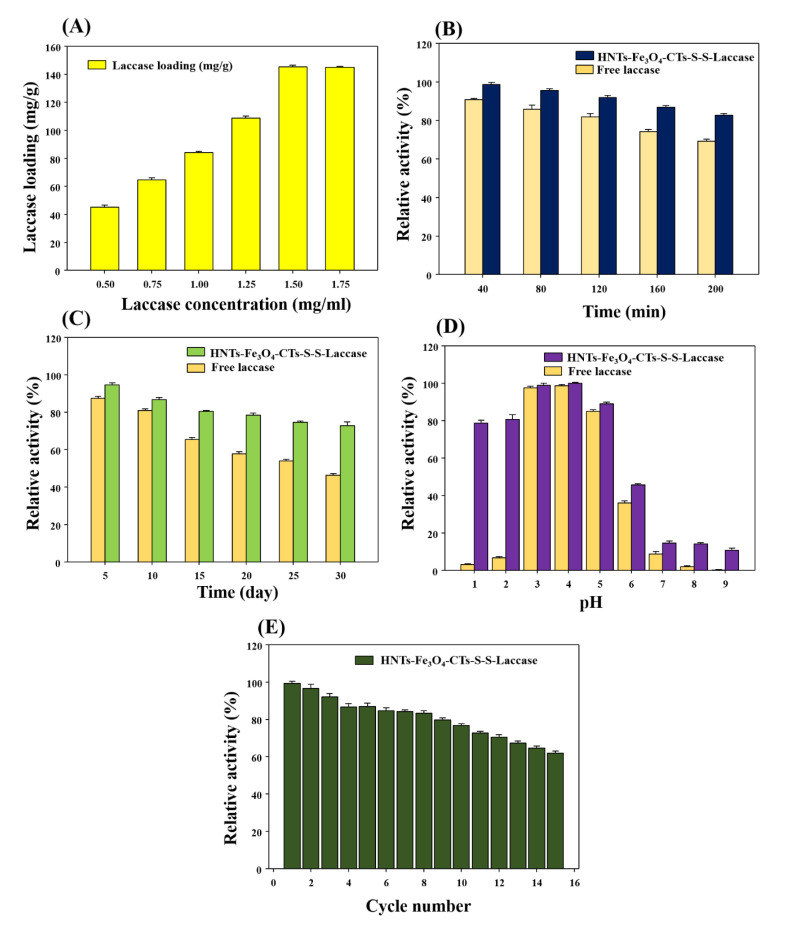
(**A**) Effect of initial laccase concentration on the immobilization on the HNTs-Fe_3_O_4_-CTs-SH, (**B**) temperature stability at 60 °C for 200 min by free laccase and HNTs-Fe_3_O_4_-CTs-S-S-Laccase, (**C**) storage stability for 30 days by free laccase and HNTs-Fe_3_O_4_-CTs-S-S-Laccase, (**D**) pH stabilities free laccase and HNTs-Fe_3_O_4_-CTs-S-S-Laccase, and (**E**) repeated cycle activities of the HNTs-Fe_3_O_4_-CTs-S-S-Laccase.

Furthermore, the effect of initial laccase concentrations on laccase loading over the surface of HNTs-Fe_3_O_4_-CTs-SH was assessed (Figure 6A). In the presence of the initial laccase concentrations of 0.5, 0.75, 1.0, 1.25, 1.5 and 1.75 mg/mL, HNTs-Fe_3_O_4_-CTs-SH displayed 45, 64, 84, 108, 145, and 144 mg/g of laccase loading. The laccase loading on HNTs-Fe_3_O_4_-CTs-SH was found to increase with a subsequent increase in the initial laccase concentrations from 0.5 to 1.5 mg/mL. The optimum laccase loading (144 mg/g) was reached 1.5 mg/mL and remained stable with a further increase in the laccase concentrations. This result indicated that the optimum occupation of the immobilization sites on HNTs-Fe_3_O_4_-CTs-SH was reached at the 1.5 mg/mL initial laccase concentration. The obtained value of the laccase loading was found to be excellent in comparison to the most recent reports (Table 1). The obtained laccase loading in this study was found to be higher in comparison with the recently reported nanosupports. This indicates that thiol functioned CTs over clay mineral HNTs with magnetic properties can be excellent supports for the enzyme immobilization.

### 3.4. Biocatalytic Performance of the HNTs-Fe_3_O_4_-CTs-S-S-Laccase

After assessment of the immobilization activity recoveries and understanding of the laccase loading pattern on HNTs-Fe_3_O_4_-CTs-SH, the biocatalytic performance of the HNTs-Fe_3_O_4_-CTs-S-S-Laccase was evaluated by using the temperature stability, storage stability, pH stability, and readability potential studies. First, the data obtained for temperature stability is presented in Figure 6B. Enzyme being proteinaceous is extremely prone to denaturation due to elevated temperatures [23]. Excellent enzyme immobilization strategies were found to be effective to overcome this problem [44]. Hence, in this study free laccase and the immobilized enzyme system were assessed for temperature stability in acetate buffer (100 mM, pH 4.2) for 200 min at 60 °C. After 200 min, free laccase lost 31% of the initial activity (Figure 6B). However, HNTs-Fe_3_O_4_-CTs-S-S-Laccase lost 17% of the initial activity (Figure 6B). Hence, the obtained results in Figure 6B suggest significantly improved thermal stability of the laccase after the immobilization protocol. There are many applications of the enzyme laccase that can be enhanced by improved thermal stability [35]. Further, the storage stability of the immobilized and free laccase was tested (Figure 6C). The long-term storage of the enzyme in solution is a challenging task [35]. However, after immobilization, the enzyme native structure can be protected for a longer time [10]. This helps the utilization of enzyme-based catalysis and makes it economically applicable [9]. In this regard, the immobilized laccase and free laccase were tested for storage stabilities. After 30 days incubation of both the systems, free laccase retained 46% of the initial activity. However, HNTs-Fe_3_O_4_-CTs-S-S-Laccase retained 73% of the activity. A significant increase in activity stability was found after the immobilization. This can be helpful to the many laccase-based applications. Moreover, the pH of the system is a major key for the biocatalysts. The effect of pH on the immobilized laccase and free laccase with their incubation for 1 h was tested (Figure 6D). The HNTs-Fe_3_O_4_-CTs-S-S-Laccase exhibited excellent biocatalysis over the range of pH of 1–9, compared to the free laccase (Figure 6D). This will elicit the laccase application profile at various pH ranges [13]. Overall all the stability experiments proved HNTs-Fe_3_O_4_-CTs-S-S-Laccase as an excellent biocatalyst and HNTs-Fe_3_O_4_-CTs-SH can be used for the immobilization of other enzymes.

The free enzyme offers only a single use, as it is a very difficult as well as costly affair to separate enzyme and products after the reaction process [10]. Hence, nanosupport with immobilized enzyme provides leverage to overcome this limitation [1]. Besides, the magnetized nanosupports would be an excellent choice in this regard [44]. The HNTs-Fe_3_O_4_-CTs-S-S-Laccase is a capable biocatalyst and super-magnetic in nature. Hence, its potential was tested for repeated use. The data for repeated use application of the HNTs-Fe_3_O_4_-CTs-S-S-Laccase is given in Figure 6E. Upon 15 cycles of repeated use, HNTs-Fe_3_O_4_-CTs-S-S-Laccase possessed 61% of the initial activity. The HNTs-Fe_3_O_4_-CTs-S-S-Laccase exhibited significant potential of reusability. This can be highly applicable in many laccase-based applications. Being a support made from clay mineral (HNTs), super-magnetic Fe_3_O_4_ NPs, and thiol functionalized biopolymer (CTs), this nanosupport can be highly beneficial in many desired applications of the laccase. Furthermore, the HNTs-Fe_3_O_4_-CTs for laccase immobilization with glutaraldehyde (GTA) cross-linking route was reported in our previous study [13]. The same backbone nanosupport HNTs-Fe_3_O_4_-CTs was used in this study for laccase immobilization through the thiolation route. Hence, it is imperative to discuss and compare both the routes for laccase immobilization. The thiolated support gave a laccase loading capacity of 144 mg/g; however, GTA cross-linking exhibited 100 mg/g of laccase loading capacity. Thiolation enhanced the laccase-loading capacity, this might be due to the enhanced disulfide linkage formation in the immobilization process. Regarding storage and pH stability, thiolated HNTs-Fe_3_O_4_-CTs gave a better biocatalytic performance than GTA cross-linked HNTs-Fe_3_O_4_-CTs [13]. Furthermore, in a comparison of the temperature stabilities the GTA cross-linked possessed slightly higher temperature stability than thiolated HNTs-Fe_3_O_4_-CTs [13]. The thiolated HNTs-Fe_3_O_4_-CTs possessed remarkable enhancement in the repeated cycle studies [13]. These comparisons indicated significantly improved nanosupport HNTs-Fe_3_O_4_-CTs-SH was provided for the laccase immobilization. This upgraded nanosupport can enable the exploration of many laccase-based applications, and it can also be applied to other biocatalyst immobilization processes, to enhance their biocatalytic performances.

### 3.5. Application of HNTs-Fe_3_O_4_-CTs-S-S-Laccase in Environmental Pollutants Removal

**Figure 7:**
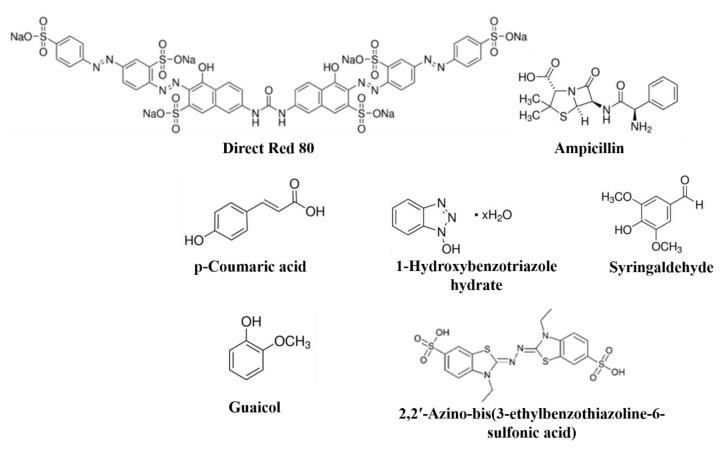
Structures of the environmental pollutants Direct Red 80 and ampicillin, and redox mediators, p-coumaric acid (CA), 1-1-hydroxy benzotriazole hydrate (HBT), syringaldehyde (SA), guaiacol (GUA), and 2,2′-Azino-bis(3-ethylbenzothiazoline-6-sulfonic acid) (ABTS).

Environmental pollutants such as textile dyes and pharmaceutical compounds cause significant damage to the natural ecosystem and human health. Synthetic reactive dyes released from the textile industry have very serious implications for water ecosystems and humans [3]. Similarly, pharmaceutical compounds released from domestic and industrial wastewater in small concentrations pose a significant challenge to the environment due to their bioactive nature [4]. Laccase catalyzes the redox-mediated degradation of various pollutants. Still, free laccase has many limitations to mitigate this challenge, however, immobilized laccase with improved properties is most suitable for redox-mediated removal of the various pollutants from water [1]. The enhanced biocatalytic performance by the immobilized laccase plays a crucial role in the degradation of environmental pollutants. Hence, in this study, we applied the developed immobilization system of HNTs-Fe_3_O_4_-CTs-S-S-Laccase for the removal of textile dye DR80 and the pharmaceutical compound ampicillin. The structures of environmental pollutants taken for study, DR80 and ampicillin, and the redox mediator compounds, such as p-Coumaric acid (CA), 1-Hydroxybenzotriazole hydrate (HBT), syringaldehyde (SA), guaiacol (GUA), and 2,2′-Azino-bis (3-ethylbenzothiazoline-6-sulfonic acid) (ABTS) are shown in Figure 7.

**Figure 8:**
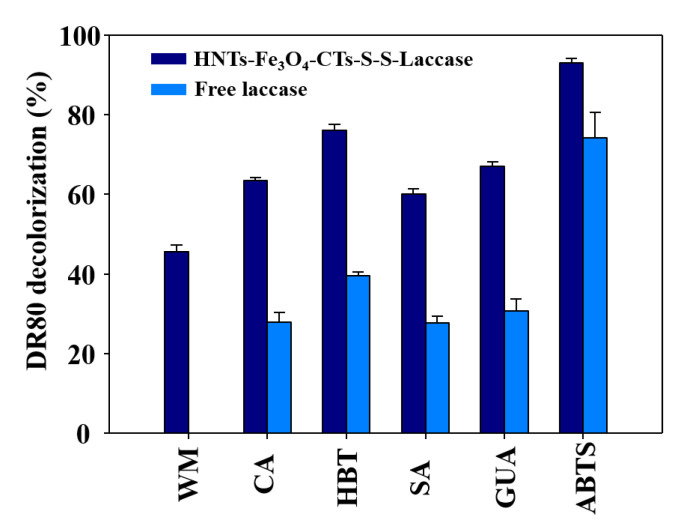
Removal of the DR80 by free laccase and HNTs-Fe_3_O_4_-CTs-S-S-Laccase; without a mediator (WM), and with redox mediators of CA, HBT, SA, GUA, and ABTS.

The HNTs-Fe_3_O_4_-CTs-S-S-Laccase and free laccase were applied for the degradation of DR80. The obtained results are shown in Figure 8. The free laccase does not show the decolorization of DR80 without a redox mediator. Laccase requires the redox mediator system to degrade the environmental pollutants [16]. The HNTs-Fe_3_O_4_-CTs-S-S-Laccase without redox mediator gave 45% removal of DR80 Figure 8. Consequently, without a mediator immobilized laccase cannot attack the DR80 directly, and obtained 45% DR80 removal in the absence of the mediator was assigned to the adsorption process. Next, HNTs-Fe_3_O_4_-CTs-S-S-Laccase with redox mediators CA, HBT, SA, GUA, and ABTS, gave 63, 76, 60, 67, and 92% decolorization of DR80 Figure 8. However, free laccase exhibited 27, 39, 27, 30, and 74% decolorization of DR80 in the presence of the CA, HBT, SA, GUA, and ABTS (Figure 8). Thus, HNTs-Fe_3_O_4_-CTs-S-S-Laccase enhanced the decolorization of DR80 compared to the free laccase. This enhancement corresponded to the enhanced catalytical performance and possible adsorption effect of the HNTs-Fe_3_O_4_-CTs-S-S-Laccase. The immobilized laccase or laccase first oxidized the mediator, and the oxidized mediator carried out the oxidative degradation of the DR80. Among all redox mediators studied, the ABTS was found to be the best for the DR80 removal potential of the HNTs-Fe_3_O_4_-CTs-S-S-Laccase. This obtained result might be due to the better oxidation−reduction potential of the ABTS by HNTs-Fe_3_O_4_-CTs-S-S-Laccase. Hence, the developed laccase immobilization system was found to be successful in the removal of a toxic textile dye like DR80.

**Figure 9:**
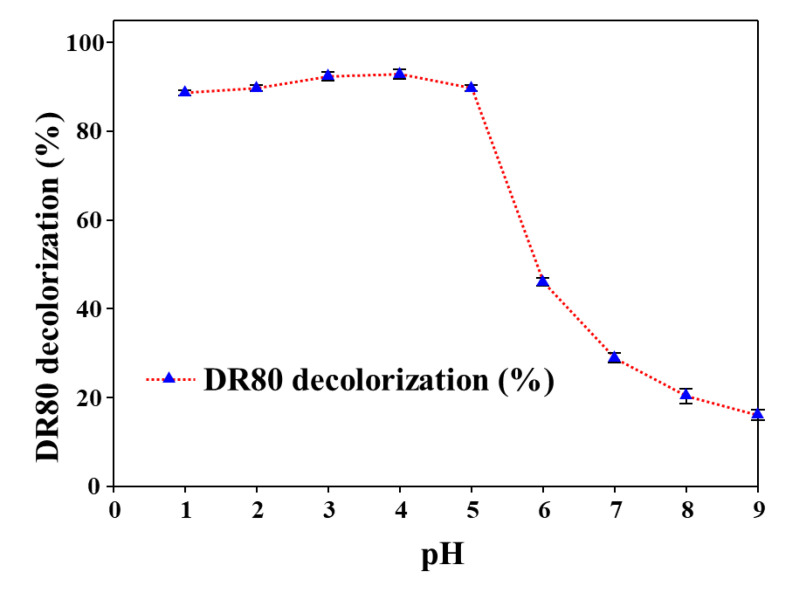
Effect of pH on removal of the DR80 by HNTs-Fe_3_O_4_-CTs-S-S-Laccase in the presence of the redox mediator ABTS.

After investigating the decolorization potential of HNTs-Fe_3_O_4_-CTs-S-S-Laccase, the effect of pH on the decolorization of DR80 in the presence of redox mediator ABTS was assessed. pH played a crucial role in biocatalytic reactions of the laccase. The HNTs-Fe_3_O_4_-CTs-S-S-Laccase exhibited higher activities over all the pH range (Figure 6D). The decolorization performance of DR80 at various pH values of 1, 2, 3, 4, 5, 6, 7, 8, and 9 was observed as follows: 88, 89, 92, 92, 89, 46, 28, 20, and 16%, respectively (Figure 9). The pH range 1–5 displayed higher decolorization. The highest decolorization was displayed at pH 4, this obtained result was in agreement with previous observations from Figure 6D.

**Figure 10:**
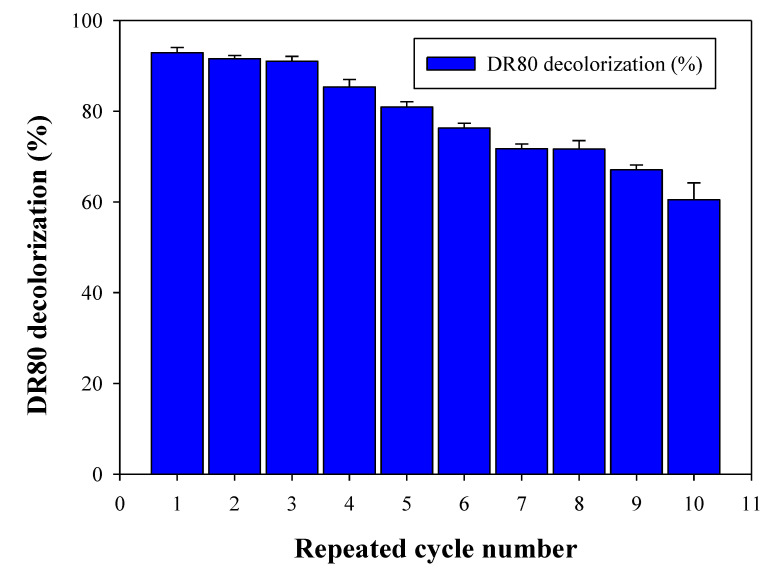
Repeated cycle removal of the DR80 by HNTs-Fe_3_O_4_-CTs-S-S-Laccase in the presence of the redox mediator ABTS.

Furthermore, the repeated cycle decolorization of the DR80 was assessed. It was very important to apply laccase in multiple cycles to enhance the remediation potential. The HNTs-Fe_3_O_4_-CTs-S-S-Laccase was assessed for 10 decolorization cycles of DR80 in the presence of redox mediator ABTS (Figure 10). The HNTs-Fe_3_O_4_-CTs-S-S-Laccase displayed 92, 91, 91, 85, 80, 76, 71, 71, 67, and 60% decolorization of the DR80. At the end of the 10th cycle, 60% decolorization was observed. This marks higher remediation potential of the developed immobilized laccase system. However, in the case of free laccase, it was very difficult to separate laccase from the first reaction mixture. However, the HNTs-Fe_3_O_4_-CTs-S-S-Laccase is super-magnetic, can be easily retrieved from the water, and applied in the next cycle of pollutant removal. The overall DR80 removal studies by HNTs-Fe_3_O_4_-CTs-S-S-Laccase gave an idea about its potential for the treatment of textile dyes.

**Figure 11:**
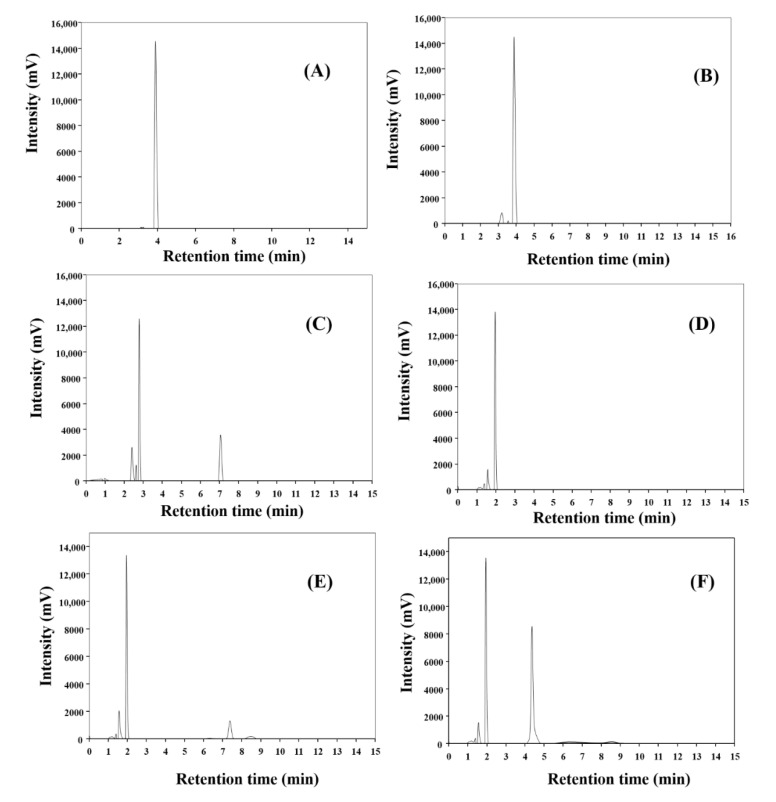
HPLC analysis of the ampicillin degradation by HNTs-Fe_3_O_4_-CTs-S-S-Laccase (**A**) control ampicillin, (**B**) ampicillin + HNTs-Fe_3_O_4_-CTs-S-S-Laccase (in absence of the redox mediator), (**C**) ampicillin + HNTs-Fe_3_O_4_-CTs-S-S-Laccase + ABTS, (**D**) ampicillin + HNTs-Fe_3_O_4_-CTs-S-S-Laccase + GUA, (**E**) ampicillin + HNTs-Fe_3_O_4_-CTs-S-S-Laccase + SA, and (**F**) ampicillin + HNTs-Fe_3_O_4_-CTs-S-S-Laccase + HBT.

Finally, the HNTs-Fe_3_O_4_-CTs-S-S-Laccase was tested for its potential in the degradation of the ampicillin. The fate of antibiotics like ampicillin in water bodies is causing serious environmental concern mainly due to the possible spread of antibiotic resistance [45]. Among all classes of antibiotics, the β-lactam class of antibiotics has captured over 65% of the world antibiotic market. Ampicillin is a widely used β-lactam antibiotics [46]. The role of laccase for degradation of the β-lactam antibiotics has been reported [47]. Hence, in this study, we assessed the potential of the HNTs-Fe_3_O_4_-CTs-S-S-Laccase for ampicillin degradation. The degradation of ampicillin by HNTs-Fe_3_O_4_-CTs-S-S-Laccase was observed by the HPLC analysis as mentioned in the report [48]. The obtained results for redox-mediated degradation of ampicillin by HNTs-Fe_3_O_4_-CTs-S-S-Laccase are shown in Figure 11. The HPLC of control ampicillin showed a very sharp peak at the retention time of the 3.9 min (Figure 11A). A similar kind of ampicillin peak was reported in earlier reports [49,50,51]. The ampicillin in the presence of the HNTs-Fe_3_O_4_-CTs-S-S-Laccase and with no redox mediator showed a very sharp peak at the retention time of the 3.9 min (Figure 11B). This obtained result corroborated that, without a mediator, no degradation was observed. The intensity of the peak also remained consistent suggesting no adsorption of ampicillin. Further, HNTs-Fe_3_O_4_-CTs-S-S-Laccase with redox mediator ABTS exhibited a changed profile of the peaks at different retention times; such as 2.4, 2.6, 2.79, and 7.07 min, respectively, see Figure 11C. This observation confirmed the complete degradation of the ampicillin. The main ampicillin peak of 3.9 min disappeared and new peaks arrived due to degradation by the HNTs-Fe_3_O_4_-CTs-S-S-Laccase. Similarly, the HNTs-Fe_3_O_4_-CTs-S-S-Laccase with mediators GUA, SA, and HBT gave degradation of ampicillin with different retention time peaks (Figure 11D–F). Thus, the overall ampicillin degradation study corroborated that various redox mediators can be used for ampicillin degradation by HNTs-Fe_3_O_4_-CTs-S-S-Laccase. Therefore, this system could be used for a wide range of pollutant removal. As in this study, we assessed the potential of immobilized laccase for textile dye DR80 and pharmaceutical compound ampicillin.

## 4. Conclusions

In conclusion, this study investigated the new nanocomposite containing HNTs, Fe_3_O_4_ NPs, and thiolated CTs for laccase immobilization. The detailed characterizations, FE-SEM, HR-TEM, XPS, XRD, FT-IR, and VSM analyses of the nanocomposite corroborated successful synthesis. The immobilized laccase displayed outstanding biocatalytic performance with improved thermal, storage and pH stability. The immobilized laccase also gave redox-mediated degradation of environmental pollutants such as DR80 and ampicillin. This indicated that immobilized laccase can be applied for wastewater treatment. The super-magnetic nature can easily retrieve the nanosupport from the solution after the decontamination of the pollutant. Thus, the novel nanosupport developed in this study “HNTs-Fe_3_O_4_-CTs-SH” is highly efficient for laccase immobilization, and also can be applied for other enzyme-immobilization processes.

## Figures and Tables

**Table 1:** Laccase loading capacity (mg/g) comparison of the recently reported nanosupports.

Used Material	Laccase Loading (mg/g)	Reference
HNTs-Fe_3_O_4_-CTs-SH	144	This study
Magnetic biochar (L-MBC)	27	[35]
MACS-NIL-Cu-Laccase	47	[36]
Polyacrylamide-alginate cryogel	68	[8]
LA-Au/PDA@SiO_2_-MEPCM	50	[37]
ZrO_2_–SiO_2_	86	[38]
Fe_3_O_4_@Chitosan	32	[39]
ZrO_2_–SiO_2_/Cu^2+^	94	[38]
HNTs-M-chitosan (1%)	100	[14]
Aminosilanized magnetic HNTs	84	[16]
Fe_3_O_4_-NIL-DAS@lac	60	[40]
Magnetized chitosan modified α-Cellulose	73	[9]
PD-GMA-Ca@ABTS beads	8	[41]
Magnetized chitosan modified HNTs	92	[13]
Chitosan microspheres	8	[42]
Sepharose-linked antibody	33	[43]
